# Dysregulation of Chemokine/Chemokine Receptor Axes and NK Cell Tissue Localization during Diseases

**DOI:** 10.3389/fimmu.2016.00402

**Published:** 2016-10-06

**Authors:** Giovanni Bernardini, Fabrizio Antonangeli, Valentina Bonanni, Angela Santoni

**Affiliations:** ^1^Department of Molecular Medicine, Sapienza University, Rome, Italy; ^2^IRCCS NEUROMED – Mediterranean Neurological Institute, Isernia, Italy; ^3^Department of Molecular Medicine, Istituto Pasteur-Fondazione Cenci Bolognetti, Rome, Italy

**Keywords:** chemokine receptors, CXCR4, CXCR3, NK cell subsets, multiple sclerosis, multiple myeloma, cross-inhibition, migration

## Abstract

Chemokines are small chemotactic molecules that play key roles in physiological and pathological conditions. Upon signaling *via* their specific receptors, chemokines regulate tissue mobilization and trafficking of a wide array of immune cells, including natural killer (NK) cells. Current research is focused on analyzing changes in chemokine/chemokine receptor expression during various diseases to interfere with pathological trafficking of cells or to recruit selected cell types to specific tissues. NK cells are a heterogeneous lymphocyte population comprising several subsets endowed with distinct functional properties and mainly representing distinct stages of a linear development process. Because of their different functional potential, the type of subset that accumulates in a tissue drives the final outcome of NK cell-regulated immune response, leading to either protection or pathology. Correspondingly, chemokine receptors, including CXCR4, CXCR3, and CX_3_CR1, are differentially expressed by NK cell subsets, and their expression levels can be modulated during NK cell activation. At first, this review will summarize the current knowledge on the contribution of chemokines to the localization and generation of NK cell subsets in homeostasis. How an inappropriate chemotactic response can lead to pathology and how chemokine targeting can therapeutically affect tissue recruitment/localization of distinct NK cell subsets will also be discussed.

## Introduction

Natural killer (NK) cells are innate lymphocytes that play a key role in the immune response to tumors and infections through their ability to kill transformed or infected cells and to produce immunoregulatory cytokines and chemokines. Activation of NK cell effector functions can be achieved through a complex integration of inhibitory and activation signals provided by membrane expressed receptors and/or through cytokine stimulation (1).

Natural killer cells are widely distributed into different tissues such as the bone marrow (BM), liver, thymus, lymph node, and uterus, thus contributing to immune surveillance in homeostasis, and can be further recruited into tissues in pathological conditions (2, 3). While tissue-resident NK cells have been identified in uterus, liver, and skin, conventional NK cells continuously traffic and localize into tissue through a combination of stimuli able to promote their mobilization from storage compartments to blood circulation and their entry and retention into tissue (4–6).

Chemokines are a family of more than 50 small proteins, mostly secreted, that accomplish their function by interacting with heterotrimeric G protein-coupled receptors (GPCR). Chemokine binding promotes a conformational change in the receptor, triggering intracellular signals that drive cell polarization, migration, and adhesion, thus resulting in the induction of leukocyte trafficking and homing (3). Besides leukocyte chemotaxis, chemokines can affect a number of other leukocyte functions and are recognized as important regulators of the immune response (3). Chemokines may be grouped according to their modality of expression and function, as inflammatory or homeostatic (7).

## Chemokine Receptor Expression on NK Cell Subsets

A number of evidence indicates that the differential functional properties underlying NK cell-mediated protective effect in pathological conditions can be attributed to distinct NK cell populations endowed with distinct expression patterns of activating and inhibitory as well as homing receptors. Two major subsets of mature NK cells were identified in human peripheral blood with respect to the neural cell adhesion molecule CD56 and the low affinity receptor for IgG, FcγRIII CD16. The CD56^high^CD16^low^ subset accounts for around 10% of circulating CD56^+^ NK cells and exerts immunomodulatory effects producing large amount of cytokines such as IFNγ in response to activation, whereas CD56^low^CD16^high^ cells are the major cytotoxic population representing the majority of circulating CD56^+^ NK cells [for a review on human NK subsets, see Ref. (8)]. Although the exact relationship between these NK cell subsets still remains unclear, evidence suggest that CD56^low^ NK cells originate from CD56^high^ NK cells (9–12).

**Figure 1:**
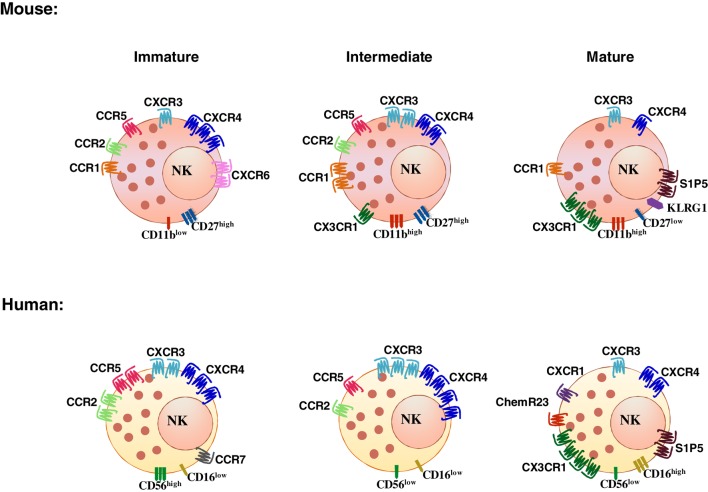
**Differential chemoattractant receptor expression by NK cell subsets in human and mouse**.

Natural killer cell subsets display a differential pattern of chemokine receptor expression (Figure 1). CD56^high^ NK cells are targeted to lymph nodes *via* CCR7, preferentially express CXCR3 and have higher CXCR4 expression levels as compared with CD56^low^ cells. CD56^low^ NK cells uniquely express CXCR1, ChemR23, and CX_3_CR1 (Figure 1) (see Table 1 for a list of ligands of chemoattractant receptors expressed by human and mouse NK cell subsets) (13–18). More recently, a new CD56^low^CD16^low^ subset has been identified and found to be prominent in the BM of healthy pediatric donors, to display potent killing and IFNγ producing capacity, and to expresses higher levels of CXCR4 and CXCR3 compared with the other subsets (17). In addition, other subsets related to NK cell maturation, including cells coexpressing CD57, a member of the glucuronyl-transferase gene family or the L-selectin within the CD56^low^ NK cells are currently active field of investigation (19–21).

**Table 1:** **Chemokine receptor expression by NK cells and their respective ligands**.

Chemokine receptor	Chemokine ligand
CCR1	CCL3/MIP-1α, CCL5/RANTES, CCL7/MCP-3, CCL9/CCL10/MIP-1γ, CCL14/HCC-1, CCL15/HCC-2, CCL16/HCC-4, CCL23/MPIF-1
CCR2	CCL2/MCP-1, CCL7/MCP-3, CCL12, CCL13/MCP-4, CCL16/HCC-4
CCR5	CCL3/MIP-1α, CCL4/MIP-1β, CCL5/RANTES, CCL8/MCP-2, CCL14/HCC-1
CCR7	CCL19/MIP-3β/ELC, CCL21/SLC
CXCR1	CXCL8/IL-8
CXCR3	CXCL9/Mig, CXCL10/IP-10, CXCL11/I-TAC
CXCR4	CXCL12/SDF-1
CXCR6	CXCL16/SR-PSOX
CX_3_CR1	CX3CL1/fractalkine

**Chemoattractant receptors**	**Ligand**

S1P5	S1P
ChemR23, CCRL2	Chemerin

*The previous chemokine name follows the one currently used*.

Mouse NK cells do not express the murine ortholog of CD56, but four different developmentally related subsets have been identified on the basis of the expression levels of the integrin chain CD11b and of a member of the TNF receptor superfamily, CD27: CD11b^low^CD27^low^, CD11b^low^CD27^high^, CD11b^high^CD27^high^, and CD11b^high^CD27^low^ (22, 23). The inhibitory receptor KLRG1 is acquired by the most mature subset and identifies NK cells with reduced effector functions (24). KLRG1 coexpression with the chemokine receptor CX_3_CR1 identifies an even later maturation stage with unique functional properties (25). Likewise, other NK cell subsets can be defined according to the expression of selected chemokine receptors: the prevalent expression of CXCR3 on CD56^high^ was related to expression of the receptor on mouse CD27^high^ NK cells and more in general to stronger proliferative and cytokine production capacity (26, 27). In addition, CXCR6 was shown to identify an NK cell population resident in liver displaying in humans a CD56^high^CD16^−^CD57^−^ phenotype, and expressing TRAIL in diseased liver, and in the mouse a DX5^−^TRAIL^+^ phenotype (28, 29).

## Chemokine Receptor Interplay for Efficient Migration in Complex Chemotactic Environment

Leukocytes express multiple chemokine receptors not only to robustly and promptly infiltrate tissues upon activation but also to navigate through the network provided by multiple competing chemotactic gradients perceived into tissue. Indeed, discrimination of a dominant chemoattractant is required to localize in the correct microenvironment and/or to make a decision on whether to stay or to leave a tissue (30, 31). The ability to preferentially respond to selected chemoattractants was at first demonstrated in neutrophils and was postulated to require heterologous receptor desensitization that is the transinhibition of a chemokine receptor resulting from the activation of second messenger-dependent kinases by a dominant chemoattractant receptor (32–34). In this regard, triggering CXCR2 by KC was shown to be required for neutrophil egress from the BM not only by promoting neutrophil migration but also by inhibiting CXCR4-mediated BM retention (35). Afterward, our group showed that CXCR4 heterologous desensitization is also associated with NK cell egress from BM into circulation (36, 37). On the other hand, the sphingophospholipid chemoattractant sphingosine-1-phosphate (S1P) promotes NK cell egress from BM under steady state without inducing CXCR4 heterologous desensitization (38). CX3CL1, a ligand for CX_3_CR1 constitutively expressed in BM, acts similarly on a small highly differentiated subset which poorly expresses CXCR4 (25, 39). While homeostatic chemokines may be sufficient for NK cell egress under steady state, heterologous receptor desensitization could be a mechanism to rapidly switch NK cell responsiveness promoted by inflammatory chemokines, to promptly facilitate BM NK cell availability in circulation.

Besides desensitization, CXCR4-mediated transinhibition can occur through other mechanisms, including receptor heterodimerization and G protein scavenging (40). In regard to heterodimerization, it is becoming increasingly clear that receptor dimers are constitutively formed, and that ligand binding to one receptor can reorganize receptor complexes thus affecting different aspects of the associated chemokine receptor activity, including ligand affinity, the activated signaling cascades, and the receptor internalization (41–43). For example, CXCR3/CXCR4-heterodimerization was shown to reduce the binding affinity of CXCR4 for its ligand (44).

Apart from the mechanism involved, cross-regulation promoted by CXCR3 was shown to be relevant in several pathological conditions in mouse disease models. For example, O’Boyle and coworkers demonstrated CXCR4 and CCR5 inhibition on T cells by using a mimetic of CXCL10, a CXCR3 ligand. The use of this mimetic in a humanized mouse air-pouch model demonstrated reduced trafficking of T cells toward synovial fluids from patients with active rheumatoid arthritis, indicating that the triggering of a single chemokine receptor can control the immune response in chronic inflammatory conditions where CXCL12 is produced at high levels (45, 46). More recently, our observations in a mouse model of multiple myeloma led us to hypothesize that increased expression of CXCR3 ligands in the tumor BM microenvironment constitutes a new mechanism to avoid tumor infiltration by NK cells: ligand-induced CXCR3 activation on KLRG1^−^ NK cells resulted in cross-desensitization of CXCR4 that together with the coincident down-modulation of CXCL12 protein levels promotes NK cell egress from BM into blood circulation. The final outcome of this process is the reduction of the localization of this subset at the tumor site (39). On the other hand, when studying human plasmacytoid dendritic cells (pDC), a positive cooperative interaction was observed between the two receptors (47). This observation, together with a consistent adjacent expression of CXCL12 with CXCR3 ligands in human tissues, led the authors to hypothesize that the cooperation between CXCR3 ligands and CXCL12 controls the tissue recruitment of pDCs.

Considering the highlighted importance of CXCR3/CXCR4 interplay, hereafter we will document the critical role of CXCR3 and of CXCR4 receptor/ligand axes in the regulation of NK cell-mediated function in pathologies (summarized in Table 2).

**Table 2:** **Influence of dysregulation of chemokine receptor/ligand axes in pathologies on NK cell subsets**.

NK cell subset	Chemokine-related alterations	Disease	Effects	Reference
CD56^low^	↑CXCR4	Neuroblastoma	Less recruitment to tumor site – tumor immune evasion	(55)
	↓CX_3_CR1			
KLRG1^−^	↓CXCL12	Multiple myeloma	Less recruitment to tumor site – tumor immune evasion	(39)
	↓CXCR3			
	↑CXCL9/10			
NK cells	↑CXCR4	Multiple sclerosis	Enhanced chemotaxis toward CXCL12 – neuroprotective role	(69)
CD56^high^	↓CXCR4	Paroxysmal nocturnal hemoglobinuria	Less secondary lymphoid tissue relocation	(58)
CD56^low^	↓CXCR4	GATA2 deficiency	Reduced chemotaxis toward CXCL12	(63)
NK cells	↑CXCR4	WHIM	Enhanced chemotaxis toward CXCL12	(38, 64, 65)
CD27^high^	↑CXCL9/10	Cowpox virus infection	Increased recruitment to lymph nodes	(77)
			Protection from infection	
NK cells	↑CXCL10	HCV infection	Less recruitment to liver – immune response dampening	(81)
	Truncated form			
CD56^low^	↑CXCL9/10	Primary biliary cirrhosis	Autologous cytotoxicity – tissue damage	(82–85)
CD56^high^	↑CXCR3	Psoriatic skin	Local inflammation – disease progression	(86)
	↑CXCL10			
CD56^high^	↑CXCR3 ligands	Periprosthetic osteolysis	Local inflammation – immunoregulatory role	(87)

*↑ indicates up-modulation/increase frequency and ↓ indicates down-modulation/reduced frequency*.

## CXCR4 Receptor/Ligand Axis

CXCL12 displays a constitutive but restricted expression pattern *in situ*, with selective expression by CXCL12 abundant reticular (CAR) cells and osteoblasts in BM, by subpopulations of neuronal and endothelial cells in the brain, by dermal endothelial cells, and by invading trophoblast cells and lymph node high endothelial venules (48–54). Correspondingly, CXCR4/CXCL12 axis was reported to regulate NK cell functions in several physiological processes. It was shown that CXCL12 regulates the positioning in the BM of selected NK cell subsets at various stages of maturation; in addition, during pregnancy, human peripheral blood CD56^high^CD16^−^ NK cells can be recruited by CXCL12 and migrate to the uterus (36, 48).

Considering the key role of CXCL12 in the localization of NK cells in BM, subversion of the CXCR4/CXCL12 axis has been hypothesized to represent a mechanism of immune evasion from NK-mediated immune surveillance in neuroblastoma and multiple myeloma (39, 55). TGF-β1 produced by neuroblastoma cell lines was shown to upregulate the surface expression of CXCR4 and CXCR3 on both CD56^high^ and CD56^low^ NK cells, while it downregulated CX_3_CR1 in the CD56^low^ subset. Increased CXCR3 and reduced CX_3_CR1 expression was observed also in peripheral blood NK cells of stage 4 neuroblastoma patients, and it may represent an attempt to avoid NK cell cytolytic subset recruitment to the tumor site, while promoting the enrichment of immature and poorly cytotoxic CD56^high^ subset in tumor leukocyte infiltrates (55, 56). Similarly, accumulation of CD56^high^ NK cells was shown to occur in several tumors and may be related to the responsiveness of this subset to a combination of tissue-expressed chemokines: by analyzing the chemokine expression pattern of various normal solid tissues, Carrega and coworkers documented that some tissues are clearly oriented to recruit CD56^high^ cells when CXCL12 is coexpressed with other chemokines (LN, colorectal, stomach, and liver tissues), while anatomic compartments with the lowest proportion of CD56^high^ within NK cells (lung and breast tissues) display a chemokine expression profile favoring CD56^low^ NK cell recruitment (57). Similarly, decreased levels of CXCL12 in BM plasma samples from a cohort of patients with active multiple myeloma as compared with a premalignant stage support the hypothesis of a reduced NK cell surveillance based on reduced BM recruitment (39).

The key role of CXCR4 expression for NK cell homeostasis is also highlighted by genetic defects responsible for altered or lost CXCR4 function. For example, a more abundant proportion of circulating GPI^−^CD56^high^ NK cells in paroxysmal nocturnal hemoglobinuria patients, a disease caused by dampened biosynthesis of glycosylphosphatidylinositol (GPI)-linked protein, was associated with reduced responsiveness of this population to the CXCR4 ligand CXCL12 (58, 59). In addition, deficiency of the transcription factor GATA2 is characterized by several hematological and non-hematological abnormalities among which NK cell cytopenia, with almost complete absence of circulating CD56^high^ subset (60). Interestingly, CD56^low^ NK cells display reduced CXCR4 surface expression levels and reduced chemotaxis to CXCL12 that was attributed decreased expression of filamin A and β-arrestin-1, two proteins regulating CXCR4 cell surface expression and endocytosis (61–63). On the other hand, NK cells from patients affected by warts, hypogammaglobulinemia, infections, and myelokathexis (WHIM) syndrome display enhanced responsiveness to CXCL12 (38, 64, 65). Similarly, NK cells from mice displaying the most common mutation of the *CXCR4* gene associated with WHIM syndrome show enhanced migration to CXCL12 that is linked to impaired CXCR4 desensitization and internalization after CXCL12 stimulation. Possibly for this reason, NK cell distribution is altered, with CD11b^low^ and CD11b^high^CD27^high^ NK cells accumulating in the BM.

Although *CXCR4* mutation in WHIM syndrome was not associated with any NK cell-related disease, a selective defect of CXCR4 internalization after CXCL12 binding underlies a new rare immune deficiency documented in two cases of disseminated *Mycobacterium avium* infection, where a marked reduction in the number of circulating NK cells as well as neutrophils and B cells was observed (66).

An altered pattern of CXCL12 expression in brain has been reported in multiple sclerosis (67, 68), suggesting the involvement of the CXCR4/CXCL12 axis in the leukocyte infiltration that characterizes this pathology. In this regard, while the interference with CXCR4/CXCL12 axis often leads to reduced NK cell protection in pathological conditions, Serrano-Pertierra and colleagues have found increased NK cell chemotaxis in response to CXCL12 in multiple sclerosis patients in the remitting phase and in clinically isolated syndrome patients with respect to relapsing multiple sclerosis patients and healthy controls. This finding has been associated with higher frequencies of NK cells expressing CXCR4 in the blood of the former patients’ cohorts (69). The enhanced NK cell migration in patients with a less active disease course supports the idea of a neuroprotective role for NK cells in multiple sclerosis (70). Unfortunately, NK cells have been studied as a whole, and the authors agree it would be of interest to analyze NK cell subsets, also considering that the size of the circulating CD56^high^ NK cell pool is significantly associated with clinical remissions and that expansion of this population is associated with amelioration of diseases in response to therapy (71–75).

## CXCR3 Receptor/Ligand Axes

CXCR3 ligands are expressed at low levels in homeostatic conditions, but their expression can be upregulated in both the hematopoietic and non-hematopoietic compartment by IFN-γ and some related cytokines. Several studies in humans and mice reveal that NK cells can promote adaptive immune response by modulating dendritic cell (DC) function and T helper cell polarization (76). This important function is linked to CXCR3-mediated NK cell recruitment into draining lymph node in several conditions. In mouse, in accordance with higher and preferential expression levels of CXCR3, the NK cell population mostly affected by CXCR3 function is the CD27^high^ subset that colonizes draining LN following DC vaccination, cowpox virus infection, and during tumor growth (77, 78). Several studies have correlated high numbers of tumor-infiltrating NK cells with a good prognosis for cancer patients and with tumor cell clearance in mouse tumor models. This has been related to the IFN-γ promotion of CXCL9 and CXCL10 production by tumor-infiltrating leukocytes, leading to the CXCR3-mediated recruitment of mouse CD27^high^ NK cells, the population of NK cells with the higher functional potential (79).

The influence of CXCR3–CXCL10 axis on NK cell function was documented also in human pathologies. Upregulation of CXCR3 ligands in multiple myeloma patients with active disease corresponded to marked down-modulation of CXCR3 expression levels by BM NK cells, an event that was linked to reduction of NK cell localization in the BM in multiple myeloma-bearing mice. In addition, high CXCL9 and CXCL10 serum levels were associated with several established prognostic parameters and predicted poor overall survival (39, 80).

CXCL10/CXCR3 axis is involved in hepatic trafficking of NK cells, which also represent an important component of the intrahepatic lymphocyte pool and has been implicated in the pathogenesis of chronic hepatitis C virus (HCV) infection. The CD56^high^CXCR3^+^ NK cells display the strongest activity against hepatic stellate cells, thus regulating liver fibrosis. Although expanded in HCV-infected patients, the CD56^high^CXCR3^+^ NK cell subset display impaired functions that may be linked to HCV-associated liver fibrosis (27). Elevated levels of CXCL10 were found in serum of patients and were predictive of the failure to respond to HCV therapy. Nevertheless, in a recent study, it has been reported that CXCL10 in the serum of HCV patients may not be biologically active, representing a truncated form that can bind to CXCR3 without signaling. In the presence of higher levels of this CXCL10 antagonist, NK cells might fail to migrate to the infected liver and accumulate instead in the peripheral circulation (81).

Chemokines play an important role in destruction of the biliary tract (82) by recruiting cells of the immune system, including NK cells. As such, liver NK cells have been reported to express the chemokine receptors CX_3_CR1 and CXCR3 (83). Among the principal chemokines involved in hepatic immune cell migration, CXCL9 and CXCL10 are both increased in serum of patients with primary biliary cirrhosis (PBC) compared with normal individuals and are preferentially expressed in the portal areas, corresponding to CD56^low^CD16^+^ NK cell liver infiltration increased numbers of CD56^+^ cells located around the destroyed small bile ducts (84, 85).

Several reports documented that CD56^high^ NK cells also infiltrate inflamed skin in a CXCL10-dependent fashion. Ottaviani et al. have shown that psoriatic keratinocytes display an enhanced capacity to produce CXCL10, and CD56^high^CD16^−^ NK cells showed an upregulation of CXCR3, in comparison to CD56^low^CD16^+^ NK cells (86). CD56^high^CXCR3^+^CCR5^+^ cells produced IFNγ after IL2 stimulation that in turn potentiates activation of keratinocytes and upregulates HLA class-I. These findings would suggest that CD56^+^ NK cells are recruited in psoriatic skin through a mechanism involving the CXCL10/CXCR3 axes and that, once in the skin, they may contribute to the disease progression by inducing local inflammation and amplifying T cell autoimmune reactivity.

Similar to psoriatic skin-infiltrating NK cells, NK cells in the synovial tissue of osteoarthritis patients are CCR5^+^CXCR3^+^. High levels of CXCR3 and CCR5 ligands present in synovial fluids after revision surgery, as well as evidence of particle-induced chemokine production by macrophages (87), suggest a mechanism for recruitment of a subset functionally corresponding to CD56^high^CD16^−^ NK cells during periprosthetic osteolysis. The majority of synovial tissue-infiltrating NK cells express a combination of surface receptors consistent with a non-cytotoxic phenotype similar to blood.

## Conclusion

The correct localization of NK cells into tissues has a fundamental role in several aspects of NK cell-mediated immune responses *in vivo*. Thus, identification of the key mediators regulating NK cell tissue recruitment is a critical step in the optimization of current cancer immunotherapy protocols or in the treatment of inflammatory diseases.

When NK cell tissue accumulation is important, the therapeutic enhancement of expression of selected chemokines that attract NK cells specifically is a valuable approach to increase the penetration and/or local activation and differentiation of NK cells at the tumor site. Ectopic expression of chemokines/chemoattractants known to preferentially attract effector lymphocytes, including CXCR3 ligands as well as CX_3_CL1 and chemerin, was shown to positively affect the antitumor nature of tumor-infiltrating lymphocytes with a large proportion of NK cells (88–91). Nevertheless, high concentrations of attracting chemokines do not always imply increased NK cell migration, as shown by Halama and coworkers in colorectal cancer tissue where NKp46^+^ NK cells are poorly infiltrated, despite high local chemokine levels (92).

An alternative and highly novel strategy to improve NK cell migration to target tissues is to promote or optimize the expression of chemoattractant receptors on NK cells to be used for adoptive immunotherapy. The expression of chemokine receptors and the corresponding NK cell chemotactic response can be modulated upon cytokine-mediated activation thus suggesting that they may better home to tumor sites where their corresponding ligands are expressed (93–96). In addition, NK cells *ex vivo* engineered to express chemokine receptors by gene transfer or by trogocytosis are under investigation for their better tissue homing and function (97–101). Conversely, a number of new clinical trials for immune-mediated diseases based on the use of chemokine receptor antagonists are ongoing and will help to understand the therapeutic potential of these important targets for NK cell-promoted pathologies (102). Finally, the emerging role of chemoattractant receptor interplay in the regulation of immune cell response may also lead to the discovery of molecules able to block chemokine receptor cross-inhibition thus allowing to unleash the full chemotactic potential of important NK cell receptors, such as CXCR4.

## Author Contributions

GB, FA, VB, and AS contributed equally to writing and critically revised the paper.

## Conflict of Interest Statement

The authors declare that the research was conducted in the absence of any commercial or financial relationships that could be construed as a potential conflict of interest.
